# Involvement of mitochondrial pathway in NCTD-induced cytotoxicity in human hepG2 cells

**DOI:** 10.1186/1756-9966-29-145

**Published:** 2010-11-09

**Authors:** Cheng Chang, You-Qing Zhu, Juan-juan Mei, Shi-quan Liu, Jun Luo

**Affiliations:** 1 Department of Gastroenterology, Zhongnan Hospital of Wuhan University, Wuhan, 430071, China; 2 Department of Oncology, Zhongnan Hospital of Wuhan University, Wuhan, 430071, China; 3 Department of pathology, Zhongnan Hospital of Wuhan University, Wuhan, 430071, China

## Abstract

**Background:**

Norcantharidin, the demethylated analog of cantharidin derived from a traditional Chinese medicine, Mylabris, has been used in the treatment of anti-cancer effects. However, the detailed mechanisms underlying this process are generally unclear. The aim of this study was to investigate the mechanism of NCTD-induced apoptosis in HepG2 cells.

**Methods:**

The cytotoxicity was measured by MTT assay for cellular viability and by flow cytometry. The mitochondrial membrane potential and reactive oxygen species production was evaluated by flow cytometry analysis. The role of caspase activities were assayed using caspase apoptosis detection kit . Western blot analysis was used to evaluate the level of Cyto-C, Bcl-2, Bax, Bid, caspase 3, -9, -8 and PARP expression

**Results:**

After treatment with NCTD, a decrease in the viability of HepG2 cells and increase in apoptosis were observed. NCTD-induced apoptosis was accompanied by an increase in ROS production, loss of mitochondrial membrane potential and release of cytochrome c(cyto-c) from the mitochondria to the cytosol and down-regulation of anti-apoptotic protein Bcl-2 levels with concurrent up-regulation in pro-apoptotic protein Bax levels. However, another pro-apoptotic molecule, Bid, showed no change in such same treatment. NCTD-increased activity of caspase 9,caspase 3 and the subsequent cleavage caspase substrate PARP were also observed. The expression levels of pro-caspase-8 were not changed after NCTD treatment.

**Conclusion:**

These results indicate that NCTD induced cytotoxicity in HepG2 cells by apoptosis, which is mediated through ROS generation and mitochondrial pathway.

## Background

Hepatoma is the sixth most common cancer worldwide. Its incidence increased rapidly and becomes the leading cause of cancer-related deaths in the world[1]. To date, chemotherapy has been the most frequently used treatment for liver cancer and other cancers. However, The toxicity of these chemotherapy medicines to normal tissues and normal cells has been one of the major obstacles to successful cancer chemotherapy. Obviously, there is an urgent need to identify new therapeutic agents for the treatment of hepatoma. Norcantharidin (NCTD) is the demethylated analog of cantharidin isolated from natural blister beetles. In China, NCTD has been used in traditional Chinese medicine for more than two thousand years. Currently it is used as an anticancer drug to treat breast cancer, lung cancer, leukemia, colon cancer, etc[2-6]. However, the signaling pathways governing apoptosis in human HepG2 cells remains unclear.

Apoptosis is an important phenomenon in cytotoxicity induced by anticancer drugs. The execution of apoptosis, or programmed cell death[7], is associated with characteristic morphological and biochemical changes mediated by a series of gene regulation and cell-signaling pathways. Recently, perturbation of mitochondrial function has been shown to be a key event in the apoptotic cascade[8]. Anticancer drugs may damage the mitochondria by increasing the permeability of the outer mitochondrial membrane, which is associated with the collapse of the mitochondrial membrane potential (Δφm), because a decline in Δφm can disturb intracellular ATP synthesis, generation of reactive oxygen species (ROS), altered mitochondrial redox ratio, translocation of cyto c to the cytosol, and degradation of caspase-3/PARP[9-12]. In this regard, we have initiated experiments aimed at characterizing the mitochondrial function of NCTD on human HepG2 cells, a rapidly proliferating and malignant cell line.

## Materials and methods

### Chemicals and Reagents

NCTD of analytical grade purity were purchased from Sigma Chemical Co.( St. Louis, USA); a stock solution (5 mg/ml) in RPMI1640(HyClone, USA) was prepared and stored at 4°C. D-Hanks' solution, penicillin, streptomycin, fetal bovine serum, and EDTA,3-[4,5-Dimethylthiazol-2-yl]-2,5-diphenyltetrazolium bromide (MTT), propidium iodide in this study were purchased from Sigma Chemical Co(St. Louis, USA). Anti-rabbit Bcl-2, Bid, Bax, cytochrome c, and β-actin antibodies and HRP-conjugated goat anti-rabbit Ig were from R&D Systems Inc (Minneapolis, USA) . Anti-caspase-3, -8, -9 and anti-PARP were purchased from blue sky Chemical Co, LTD (Nantong, China). Dichlorodihydrofluorescein diacetate (DCHF-DA), N-acetyl-*L*-cysteine (NAC) and JC-1 kit were purchased from keygen Biotechnology Co., LTD(Nanjing, China). Caspase apoptosis detection kit and Annexin V-FITC kit were obtained from Beijing Biosea Biotechnology Co, LTD (Beijing, China).

### Cell Line and Cell Culture

The human hepatoma cell lines HepG2 was obtained from department of oncology, Zhongnan Hospital of Wuhan University (Wuhan, China), cells were cultivated in 5% CO_2 _at 37°C in RPMI1640 medium supplemented with 10% heat-inactivated fetal bovine serum, glutamine (2 mmol/L), and antibiotics (100 U/ml penicillin, 100 mg/ml streptomycin).

### Cell Viability Assay

The inhibition of cell proliferation by NCTD was determined by assaying the reduction of MTT to formazan. After incubation with NCTD for 24, 36 and 48 h, the cells(10^4^/well) in 96-well plates were washed twice with phosphate-buffered saline (PBS), and MTT (100 μg/0.1 mL of PBS) was added to each well. The cells were incubated at 37°C for 4 h, and DMSO (100 μL) was added to dissolve the formazan crystals. The absorbance rate of each well optical density (OD value) was measured at 570 nm by a spectrophotometer. The cell proliferation inhibition rate was calculated as 1-(average OD value of wells with administered drug/average OD value of control wells)×100. To explore the possibility that NCTD induced intracellular ROS in antiproliferation, the HepG2 cells were pretreated with NAC (10 mM) 2 h before treatment with NCTD, followed by NCTD (5,10,20,40 μg/ml) treatment for 24 h. HepG2 cells proliferation response was determined by MTT assay as described above. The experiments and all the below assays were repeated thrice.

### Annexin V/PI Staining Assay

To quantify the percentage of cells undergoing apoptosis, we used Annexin V-FITC kit. HepG2 cells were incubated for 24 h with NCTD (10,20,40 μg/ml). Then the cells were washed twice with cold PBS and resuspended in binding buffer at a concentration of 1 × 10^6 ^cells/ml. After incubation, 100 μl of the solution was transferred to a 5 ml culture tube, and 5 μl of Annexin V-FITC and 10 μl of PI were added. The tube was gently vortexed and incubated for 15 minutes at room temperature in the dark. At the end of incubation, 400 μl of binding buffer was added, and the cells were analyzed immediately by flow cytometry. Flow cytometry analysis was performed using the Cell Quest software.

### Analysis of ROS production

The intracellular ROS level was detected by flow cytometry using DCHF-DA.

DCHF-DA is a stable fluorescent ROS-sensitive compound, which readily diffuses into cells. DCHF-DA is hydrolyzed by esterase to form DCHF within cells, which is oxidized by hydrogen peroxide or low-molecular-weight peroxides to produce the fluorescent compound 2',7'-dichlorofluorescein(DCF). In the present study, HepG2 cells were treated with NCTD (10, 20, 40 μg/ml) for 6 h, followed by staining with DCHF-DA (100 μM) for an additional 30 min. Green fluorescence in cells under different treatments was analyzed by flow cytometry analysis. NAC(10 mM) was added 1 h prior to the treatment with 20 μg/ml NCTD for 6 h.

### Measurement of Mitochondrial Membrane Potential(Δφm)

The loss of Δφm was monitored with the dye JC-1. JC-1 is capable of selectively entering mitochondria, where it forms monomers and emits green fluorescence when Δφm is relatively low. At a high Δφm, JC-1 aggregates and gives red fluorescence. The ratio between green and red fluorescence provides an estimate of Δφm that is independent of the mitochondrial mass. Briefly, HepG2 cells (1 × 10^6 ^cells/ml) in 10-cm culture dishes were treated without or with NCTD (10,20,40 μg/ml) for 24 h. Cells were trypsinized, washed in ice-cold PBS, and incubated with 10 mM JC-1 at 37°C for 20 min in darkness. Subsequently, cells were washed twice with PBS and analyzed by flow cytometry. Excitation wave was set at 488 nm and the emitted green fluorescence of Annexin V-FITC (FL1) and red fluorescence of PI (FL2) were collected using 525 and 575 nm band pass filters, respectively.

### Detection of Cytochrome c Release from the Mitochondria to the Cytosol

Cytochrome c determination in cytosolic and mitochondrial fractions was done by western blotting. The cells were harvested without or with NCTD (10,20,40 μg/ml) for 24 h and then washed once with ice-cold PBS. For isolation of mitochondria and cytosol, the cells were sonicated in buffer containing 10 mM Tris-HCl pH 7.5, 10 mM NaCl, 175 mM sucrose, and 12.5 mM EDTA and the cell extract centrifuged at 1000 g for 10 min to pellet nuclei. The supernatant thus obtained was centrifuged at 18000 g for 30 min to pellet the mitochondria and purified as previously described. The resulting supernatant was termed the cytosolic fraction. The pellet was lysed and protein content estimated in both fractions by Bradford's method. Equal amounts of protein were separated on 15% sodium dodecyl sulfate-polyacrylamide gel electrophoresis (SDS-PAGE) then were electrotransferred to polyvinylidene difluoride (PVDF) membrane. The membrane was then incubated in 5% non-fat milk in TBST (TBS: Tris-buffered-saline, 10 mM Tris, 150 mM NaCl, pH 7.6 with 0.1% Tween 20) for 2 h followed by overnight incubation with the primary antibody separately. The incubated membranes were extensively washed with TBST before incubation for 2 h with the secondary anti-body. After extensive washing with TBST, the immune complexes were detected by enhanced chemiluminescence detection kit.

### Caspase activity assay

Analysis of caspase-3, and caspase-9 activities was performed using Caspase Apoptosis Detection Kit according to the manufacturer's instruction. In brief, after treatment with NCTD (10,20,40 μg/ml) for 24 h, cells (1 × 10^6^) were pelleted by centrifugation, washed with PBS two times and incubated in 500 μL lysis buffer on ice for 10 min, then 1 × reaction buffer and 10 μL caspase-3( DEVD-AFC), caspase-9 (IEVD-AFC)substrates was added to lysis buffer. The reaction mixtures were incubated at 37°C for 60 min. Activities of caspase-3 and -9 were measured by spectrofluorometry.

### Western blot analysis

To detect the effects of NCTD on protein expressions, we used the Western blot analysis as described in the method of Sang-Heng Kok et al [13]. After treatment with NCTD (10,20,40 μg/ml) for 24 h, the floating and adherent cells were harvested and lysed in lysis buffer (20 mM Tris-HCl at pH 7.4, 150 mM NaCl, 0.5% NP-40, 1 mM EDTA, 50 μg/ml leupeptin, 30 μg/ml aprotinin, 1 mM phenylmethylsulfonyl fluoride, PMSF). Cell lysates were then clarified by microcentrifugation at 12,000 g for 10 min at 4 °C. Aliquots (30 μg) of the cellular lysates were subjected to 12.5% sodium dodecyl sulfate-polyacrylamide gel electrophoresis (SDS-PAGE) and transferred onto a nitrocellulose membrane (Amersham Biosciences, UK). The membrane was then probed with primary antibodies to Bcl-2, Bax, Bid, cytochrome c, caspase-3, -8, -9, PARP and β-actin overnight, followed by the addition of goat anti-mouse or anti-rabbit horseradish peroxidase-link secondary antibodies. After washing, the ECL chemical reagents were added to the membrane and chemilluminescence was visualized using an enhanced chemiluminescence detection kit (Amersham, Aylesbury, UK). β-actin was used as internal control to confirm that the amounts of protein were equal.

### Statistical analysis

Data were expressed as means ± SD and analyzed using SPSS 13.0 software. Differences between the groups were evaluated by the t-test and inter-group differences were evaluated by a one-way ANOVA. *P *< 0.05 were considered statistically significant

## Result

### Proliferation inhibitory effect of NCTD

**Figure 1:**
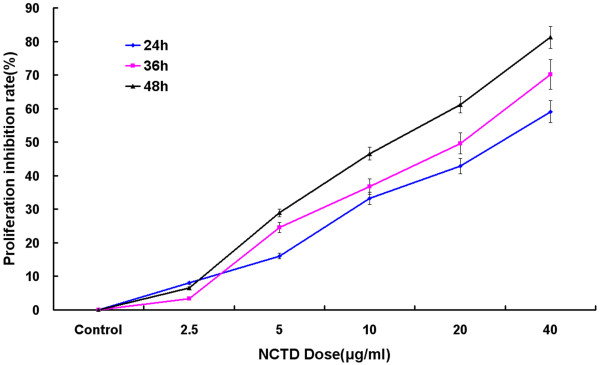
**Proliferation inhibitory effect of NCTD**. Cells were incubated with different concentrations of NCTD for 24, 36, 48 h, followed by MTT assay. As shown in Fig.1, NCTD inhibits the proliferation and cell viability of HepG2 cells in a dose-and-time dependent manner.

The inhibition of proliferation by NCTD in the human hepatoma cell HepG2 cell line was assessed after 24, 36, 48 h of drug exposure, following 24 h culture in drug-free medium. As shown in Figure 1, after treatment with NCTD, the growth inhibitory effect of NCTD at low concentrations(2.5 μg/ml) on HepG2 cells was not obvious; but as concentration increased, proliferation of HepG2 cells was markedly inhibited by NCTD in dose- and time-dependent manners at the concentrations of 5-40 μg/ml for 24, 36 and 48 h, respectively in vitro (P < 0.05).

**Figure 2:**
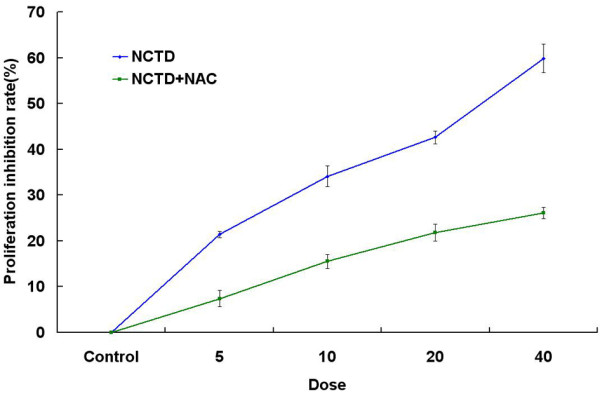
**Effect of NCTD and NCTD+NAC on HepG2 cell growth**. To explore the possibility that NCTD induced intracellular ROS in antiproliferation, the HepG2 cells were pretreated with NAC (10 mM) 2 h before treatment with NCTD, followed by NCTD (5,10,20,40 μg/ml) treatment for 24 h.

To explore the possibility that NCTD induced intracellular ROS in antiproliferation, the HepG2 cells were pretreated with NAC(10 mM) 2 h before treatment with NCTD (5,10,20,40 μg/ml) for 24 h. As shown in Figure 2 there were significant differences between NCTD and NCTD+NAC groups(*P *< 0.05)

### Flow cytometric estimation of NCTD induced apoptosis

**Figure 3:**
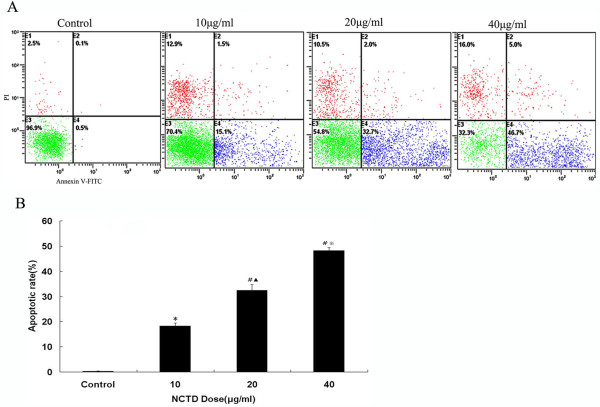
**Apoptosis Induced by NCTD**. **(A) **HepG2 cells were treated without or with NCTD (10,20,40 μg/ml) for 24 h, then processed for Annexin V/PI staining and analyzed by flow cytometry. Annexin V-positive/PI-negative cells are in early stages of apoptosis and double positive cells are in late apoptosis (B) *P < 0.05 vs Control, ^#^P < 0.01 vs Control, ^▲^P < 0.05 vs 10 μg/ml NCTD, ^※^P < 0.05 vs 20 μg/ml NCTD

Exposure of phosphotidyl serine on the surface of cells is an early event in the onset of apoptosis, which has strong binding affinity for AnnexinV in the presence of calcium HepG2 cells were incubated with differen concentration of NCTD and cells were stained with AnnexinV-FITC and PI to assess the apoptotic and necrotic cell population (Figure 3A). NCTD produced dose-dependant increase in the apoptotic cell population. The basal apoptotic population in the untreated culture was 0.3 ± 0.1%. After treatment with NCTD (10, 20, 40 μg/ml) for 24 h, the apoptotic rate raised to 18.23 ± 1.19%, 32.5 ± 2.30%, 48.23 ± 1.17% (Figure 3B), respectively.

### Generation of ROS in HepG2 cells treated with NCTD

**Figure 4:**
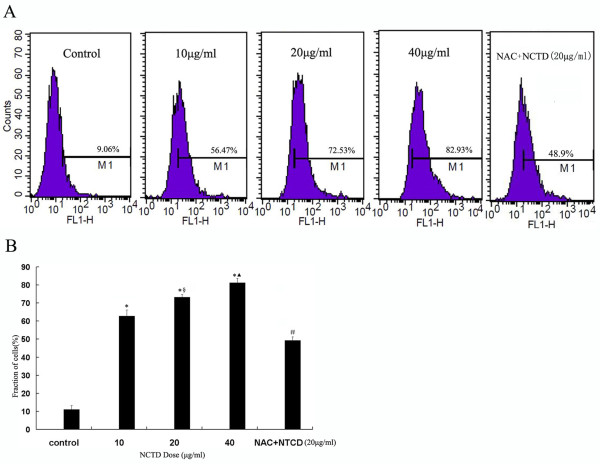
**Effect of NCTD on ROS generation in HepG2 cells**. **(A) **Cells were treated with NCTD for 6 h, followed by staining with DCHF-DA (100 μM) for an additional 30 min. NAC(10 mM) was added 1 h prior to the treatment with 20 μg/ml NCTD for 6 h.Cells treated with NCTD showed a dose-dependent increase in ROS generation. The horizontal axis represents DCFH-DA fluorescence and the vertical axis represents cell count. (B) *P < 0.01 vs Control, ^§^P < 0.05 vs 10 μg/ml NCTD, ^▲^P < 0.05 vs 20 μg/ml NCTD, ^#^P < 0.01 vs 20 μg/ml NCTD

ROS generation was analyzed by flow cytometry. Cells were treatment with various concentrations of NCTD (10, 20, 40 μg/ml) for 24 h, and then DCF fluorescence was recorded as a measure of intracellular ROS. As shown in Figure 4A, the treatment of HepG2 cells with NCTD resulted in a dose-dependent increase in ROS generation. As shown in Figure 4B, the result demonstrated that the NAC pretreated cells reduced levels of FL-1 fluorescence of DCF.

### Mitochondria Membrane Potential (Δφm) Determination

**Figure 5:**
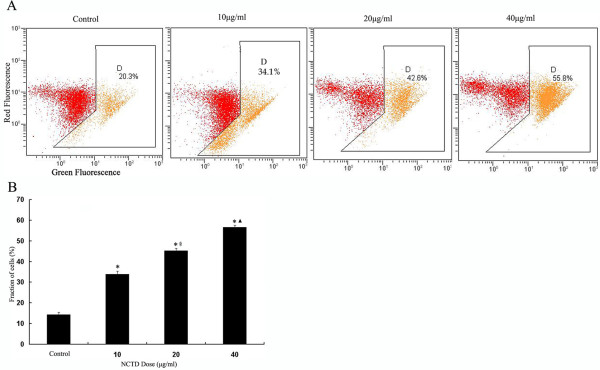
**NCTD-Induced Δφm Depolarization in HepG2 Cells**. **(A) **Cells were treated without or with NCTD for 24 h at the concentrations indicated. Change in Δφm was determined by flow cytometric analysis with JC-1. (B) *P < 0.01 vs Control, ^§^P < 0.01 vs 10 μg/ml NCTD, ^▲^P < 0.01 vs 20 μg/ml NCTD .

Disruption of mitochondrial integrity is one of the early events leading to apoptosis. To assess whether NCTD affects the function of mitochondria, potential changes in mitochondrial membrane were analyzed by employing a mitochondria fluorescent dye, JC-1. As shown in Figure 5, exposure to NCTD for 24 h resulted in a significant decrease in the ratio between red and green fluorescence by approximately 33.83 ± 1.53%, 45.23 ± 0.78%, and 56.6 ± 0.85% at 10, 20 and 40 μg/ml, respectively. This suggests that treatment with various concentrations of NCTD (10, 20, 40 μg/ml) for 24 h resulted in significant decreases of Δφm. The results imply that NCTD induces Δφm dissipation in a concentration-dependent manner.

### Cytochrome c Release from Mitochondria to Cytosol

**Figure 6:**
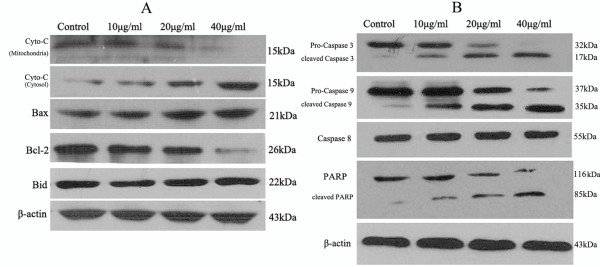
**Effect of NCTD on Expression of Cyto-C, Bax/Bcl-2/Bid, **c**aspase-3/-8/-9 and PARP proteins in HepG2 Cells**. Cells were treated with increasing doses of NCTD for 24 h. Equal amounts of whole cell extracts were fractionated by SDS-PAGE and protein were detected by Western blot analysis. **(A) **Cyto-c, Bax, Bcl-2, Bid **(B) **Caspase 3, -9, -8, PARP.

Cytochrome c release from mitochondria is a critical step in the apoptotic cascade since this activates downstream caspases. To investigate the release of cytochrome c in NCTD-treated HepG2 cells, we conducted western blotting in both the cytosolic and mitochondrial fractions. The results demonstrate a concentration-dependent increase in the cytosolic cytochrome c after treatment with NCTD. Simultaneously, there was a decrease in cytochrome c in the mitochondrial fraction (Figure 6A).

### Roles of members of the Bcl-2 family protein in NCTD-induced apoptosis

Since translocation of Bcl-2 families fromthe cytosol to the mitochondria is known to play a key role in mitochondrial-mediated apoptosis induced by a variety of apoptotic stimuli, we investigated the altered expression levels of the members of Bcl-2 family proteins such as, Bcl-2, Bax and Bid. We observed that the expression of pro-apoptotic Bax was increased in the mitochondrial fraction (Figure 6A). However, another pro-apoptotic molecule, Bid, showed no change in such same treatment. Conversely, the anti-apoptotic protein Bcl-2 was decreased in a dose-dependent manner (Figure 6A). These results suggest that NCTD might induce apoptosis through Bcl-2/Bax, but not Bid, -mediated mitochondrial dysfunction pathway

### Activation of caspase-9/caspase -3, PARP, but not caspase-8, is involved in NCTD-induced apoptosis

Since caspases are known to play a central role in mediating various apoptotic responses, we investigated which caspases are involved in NCTD-induced apoptosis of HepG2 cells. We first examined whether NCTD affects the activation of pro-caspase-8 in HepG2 cells. The expression levels of pro-caspase-8 were not changed after NCTD treatment (Figure 6B). We observed that the processing of pro-caspase-9 to active caspase-9 was increased by the treatment of NCTD in a dose-dependent manner (Table 1 & Figure 6B). We also found that NCTD significantly increased the cleavage of pro-caspase-3 to the active form in a dose-dependent manner (Table 1 & Figure 6B). Subsequently, the presence of activated caspase-3 is further confirmed by detecting the degradation of PARP, a DNA repair enzyme, which undergoes cleavage by caspase-3 during apoptosis. In NCTD -treated cells, the cleavage of PARP also occurred in a dose-dependent manner (Figure 6B).We could confirm that caspase-3 activity was also increased in a dose-dependentmanner (Figure 6B). These results suggest that NCTD -induced apoptosis is associated with the activation of caspase-9 caspase-3 and PARP but not with caspase-8.

**Table 1:** Effects of NCTD on the activation of caspase-3, -9

	Caspase 3	Caspase 9
Control	10.07 ± 1.13	36.32 ± 4.39
10 μg/ml	18.76 ± 1.22*	48.87 ± 1.72*
20 μg/ml	35.71 ± 2.83**	53.89 ± 2.54**
40 μg/ml	37.32 ± 1.28**	55.92 ± 3.16**

*P < 0.05 vs Control **P < 0.01 vs Control

## Discussion

Hepatoma remains a major public health threat and the third most common cause of death from cancer. To date, chemotherapy and radiotherapy are the most frequently used palliative treatment for liver and other cancers. However, some normal cells are destroyed as well by this method of treatment . Therefore to find novel natural compounds with low toxicity and high selectivity of killing cancer cells is an important area in cancer research. Due to the wide range of biological activities and low toxicity in animal models, some natural products have been used as alternative treatments for cancers including liver cancer.

The Chinese herb Norcantharidin (NCTD) has been used in traditional Chinese medicine for more than two thousand years. The first recorded use of cantharidin as an anti-cancer agent was in 1264[2]. Currently, multiple studies in vitro and in vivo have shown that NCTD was cytotoxic to various types of tumor cells .The significant apoptotic effects was also observed in tumor cells treated by NCTD.

Apoptosis can be initiated via two alternative signaling pathways: the death receptor-mediated extrinsic apoptotic pathway and the mitochondrion-mediated intrinsic apoptotic pathway[13-15]. Mitochondria play critical roles in the regulation of various apoptotic processes including drug-induced apoptosis[16].The mitochondrial death pathway is controlled by members of the Bcl-2 family, which play a central regulatory role to decide the fate of the cells via the interaction between pro- and anti-apoptotic members[17,18].The Bcl-2 family consists of pro-apoptotic and anti-apoptotic members[19].During apoptosis, Bcl-2 family pro-apoptotic proteins including Bim, Bax and Bid can translocate to the outer membrane of mitochondria, promote the release of pro-apoptotic factors, and induce apoptosis. On the other hand, Bcl-2 family anti-apoptotic proteins including Bcl-2 and Bcl-XL, sequestered in mitochondria, inhibit the release of pro-apoptotic factors and prevent apoptosis. When interacting with activated pro-apoptotic proteins, the anti-apoptotic proteins lose inhibiting ability of pro-apoptotic factors' release, and again promote apoptosis. Alteration in the levels of anti- and pro-apoptotic Bcl-2 family proteins influences apoptosis[20]. In this study, the NCTD-induced apoptosis in HepG2 cells was accompanied by up-regulation of Bax and the down-regulation of Bcl-2, suggesting that NCTD induced apoptosis in HepG2 cells by modulating Bcl-2 family proteins.

Recent data indicate that caspases play a key role in the initiation of apoptosis[21,22]. In the present study, NCTD treatment caused the activation of caspase-3 and -9 in a dose-dependant manner that is consistent with the results of PARP activation and cell apoptosis. These results demonstrated that NCTD-induced apoptosis may involve a caspase-3-mediated mechanism and activation of caspase-9 may act upstream of caspase-3 activation. Mitochondria have been reported to play a critical role in the regulation of apoptosis[23,24]. Consistent with these results, in the cytosol of NCTD -treated HepG2 cells, cyto c was detected after a 24 h treatment period. Once released into the cytosol, cyto c binds with procaspase-9 in the presence of ATP and Apaf-1 to form the apoptosome. This complex activated caspase-9, which, in turn, cleaves, and thereby activates, caspase-3. In NCTD-treated cells, the release of cyto c from the mitochondria was followed by activation of caspase-9 and caspase-3. It has been reported that the release of cyto c appears to be dependent on the induction of mitochondrial permeability transition, which is associated with a decrease in Δφm; therefore, the loss of Δφm and the release of apoptogenic factors, such as cyto c, from the mitochondria into the cytosol are associated with apoptosis induced by chemotherapeutic drugs[25-27]. In the present study, loss of Δφm and release of Cyto c were observed in NCTD-treated cells, resulting in caspase-9 and caspase-3 activation and PARP cleavage and, finally, apoptosis. Moreover, the loss of Δφm may, in fact, be a consequence of massive cytochrome c release from the mitochondria. Thus, a mitochondrial damage-dependent pathway may be involved in NCTD-induced apoptosis in HepG2 cells.

Some studies have reported that ROS act as secondary messengers in apoptosis induced by anti-cancer and chemopreventive agents[28,29]. The generation of ROS can cause the loss of Δφm, and induce apoptosis by releasing pro-apoptotic proteins such as AIF and Cyto c from mitochondria to the cytosol .The generation of ROS may contribute to mitochondrial damage and lead to cell death by acting as an apoptotic signaling molecule[30,31]. To reveal if NCTD influenced the level of ROS, we stained drug treated cells with DCFH-DA. We found that, in addition to its effect on Δφm, NCTD caused an increase in ROS production in HepG2 cells. The NCTD -induced increase in ROS and antiproliferation in HepG2 cells are apparently dependent on ROS generation, because the NCTD -induced increase in ROS can be

abolished or attenuated by antioxidants, such as NAC. In addition, we found that NCTD -induced antiproliferation in HepG2 cells was also abolished by the antioxidant NAC.

## Conclusions

**Figure 7:**
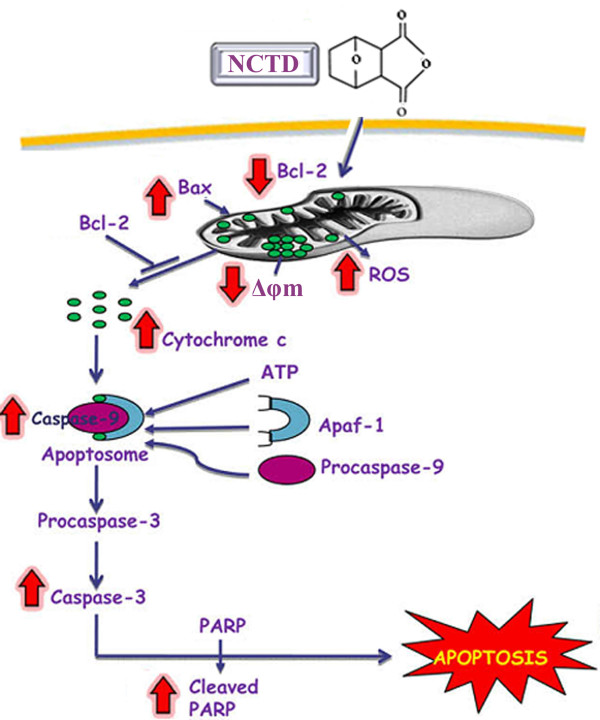
**A proposed model showing the mechanism of NCTD anti-proliferative and apoptosis effects in HepG2 cells**. ROS, reactive oxygen species; PARP, poly (ADP ribose)polymerase; Δφm, mitochondrial membrane potential; Apaf-1, apoptotic protease activating factor-1.

In conclusion, our data indicate that NCTD induced apoptosis in HepG2 cells via ROS generation and mitochondrial pathway (Figure 7)[32]. These findings suggest that NCTD may one day be used in the prevention and treatment of cancer.

## Competing interests

The authors declare that they have no competing interests.

## Authors' contributions

CC participated in research design, the writing of the paper, the performance of the research and drafted the manuscript. YQZ participated in research design, the writing of the paper and data analysis. JJM participated in the performance of the research, analysis and drafted the manuscript. SQL participated in research design and carried out the cell culture. JL provided the study concept and participated in its design and coordination. All authors read and approved the final manuscript.
